# Periscope of Dermatology

**Published:** 1898-10-29

**Authors:** 


### Periscope of Dermatology.

Lupus.—Lang[1] in a treatise on lupus, after a full description of the pathology and the symptoms of the various different stages of the disease, sums up the results of his experience in its treatment. The results obtained by such methods as scraping and scarification are, under favourable conditions, sufficiently satisfactory; but for more extensive cases the author strongly advises excision with subsequent Thiersch's grafts. When dressing the wound after grafting, instead of applying protective, he applies an aseptic dressing direct to the wound, and experiences no difficulty from adhesion of the grafts to the dressing. An analysis of his cases treated by excision and Thiersch grafting shows that of 35 patients who remained under observation, 24 were absolutely free from recurrence (in two cases during a period of four years). In ten cases recurrence took place at the seat of the operation, and in four cases in positions other than this. Some of the patients had, however suffered from lupus for a long time, even as long as 48 years.

Jones[2] recommends the excision of all cases of lupus when at all possible. In small patches, he applies Thiersch's grafts, but in more extensive cases he has found so much contraction to follow Thiersch's grafts that he cannot recommend their use. He, therefore, covers the raw surface by transplanting a partially detached skin flap raised from near to the patch. He has adopted this method in three cases, all of which did well, and did not suffer from any cicatricial contraction. Schiff[3] reports two cases of lupus treated by means of the Röntgen rays. The first case was a girl, aged 14, who had a lupus of the skin of the left forearm since the age of three. The diseased parts were exposed to the rays daily for two hours at a time. On the tenth day the first reaction appeared; the exposed parts became red and swollen, especially about the lupus infiltration, an abundant secretion trickling out from beneath the crusts; the crusts then fell off, and the lupus nodules fell out, leaving shallow, indolent, sharply-defined ulcers, surrounded by a rosy halo. At the height of the reaction (the eighteenth day), the treatment was discontinued. The epidermis of the hand and forearm fell off, and the nodules fell out. Cicatrisation proceeded slowly and at the end of two and a half months it was not completed. On the flexor surface, where the rays did not reach the skin, the lupus infiltrate remained unchanged. Under the treatment nodules became visible where none had been noticed previous to the exposure to the rays. The second case, one of lupus in the region of the larynx, reacted in the same manner as the first.

Asselbergs,[4] having treated 25 cases of lupus by injections of calomel, believes that their action on true lupus is very beneficial. All the cases treated improved, and in some there had been a complete disappearance of the lupic elements. The influence is most marked after the first injection, and the infiltrations and ulcerative processes are most affected. Cases of old ulcerative, turgescent, and deeply infiltrated lupus are most amenable to the treatment. In erythematous lupus, erythemato-tuberculosis, and tuberculosis non-exedens there is less hope from this remedy. The action of the injections upon the tuberculous nodules is uncertain. In many of his cases the cure was complete, but in others persistent nodules had to be removed by the galvano-cautery, &c., and in two cases fresh foci appeared after the cessation of the treatment. Certain cases, where ordinary treatment has failed, can be greatly benefited, if not cured. Other cases require a mixed treatment, viz., injections and cauterisations combined.

Gilchrist and Stokes[5] report a case of extensive cutaneous disease, due to the invasion of the skin by a blastomyces. The patient was a man aged 33 years. The disease began as a pimple, which soon became pustular, at the back of the left ear, and extended slowly until it covered almost the entire face. A month later a similar lesion appeared on the back of the hand, and others appeared on the scrotum, the thighs, &c.; these all healed spontaneously, leaving atrophic cicatrices. Sections of the nodules showed budding blastomycetes, which were found mostly outside the cells, but a few were found in the giant cells. Inoculations on dogs, horses, &c., produced nodules in the lungs in which numerous parasites, similar to those obtained from the patient, were found. The authors propose the name "blastomycetic dermatitis" for the disease produced by this organism.

Wells[6] also reports a case of pseudo-lupus due to blastomyces. The patient was a well-nourished man, aged 49 years, and the disease had commenced 11 years before as a small pimple on the little finger; from this it slowly spread. During the extension, the older portions would heal up, leaving a thin and weak cicatrix. The case was diagnosed as lupus, and the diseased skin was removed, the surface being covered by a plastic operation. On microscopic examination no tubercle bacilli were found, but blastomyces were discovered in the miliary abscesses, and in the giant cells.

Hallopeau[7] considers that lupus erythematosus is connected with an elementary form of tuberculosis distinct from that produced by the Koch's bacillus. He describes two forms of the disease; the acute disseminated form, and the chronic; the latter he subdivides into (1) the flat variety; (2) the hypertrophic; and (3) the asphyxial variety or lupus pernio. With regard to the prognosis, he considers it very grave in the acute cases, since about one half the cases are fatal; the acute form is, however, less persistent than the chronic form. In the chronic variety the mortality is placed at about one-sixth, but this is probably too low, since patients usually die from pulmonary consumption. With regard to treatment, it is necessary to destroy the infective agent, and to render the skin an unfavourable medium for its development. In acute cases the parts should be enveloped in compresses saturated with 1 in 5,000 sublimate solution. In the chronic cases the parts should first be washed with soft soap, and then a plaster containing salicylic acid and creosote should be applied. He has had good results from pyrogallic ointment, but its use requires care. Hutchinson[8] records a case of lupus erythematosus presumably cured by the continued application of pure carbolic acid. The acid was painted on over the edges of the patches once or twice a week, and boric acid ointment applied daily, especially after using the acid. Minim doses of Pearson's solution of arsenic were given with nux vomica and orange peel, at the same time. He considers that carbolic acid is the safest form of caustic to use for patients with lupus, &c., who are not under close observation.

[1] Brit. Jour., of Derm, May, 1898, p. 175. [2] Treatment, Mar. 24, 1898, p. 33. [3] Amer. Jour. Med. Sci., Feb , 1898, p. 236. [4] Ann. de Derm. et de Syph., Vol. 10, No. 1, p. 10; Brit. Jour. of Derm., June, 1898, p. 211. [5] Amer. Jour. Med. Sci., June, 1898, p. 737; Jour. of Exper. Med., 1898, No. 1. [6] New York Med. Jour., Mar., 1898, p. 427. [7] La Semaine Med., Mar. 18, 1898, p. 225. [8] Arch. of Surg., Jan., 1898, p. 84; Treatment, April 14, 1898, p. 86.

